# Comparative analysis of radiation therapy plans before and after biodegradable hydrogel (SpaceOAR) injection for reducing rectal toxicity in patients with prostate cancer undergoing carbon ion radiotherapy

**DOI:** 10.3389/fonc.2026.1805204

**Published:** 2026-04-20

**Authors:** Hyun Jin Kim, Hojin Kim, Sunmi Kim, Wonseuk Jang, Jin Sung Kim, Hee Kyo Jeong, Jeong Hoon Jang, Seyjoon Park, Ik Jae Lee

**Affiliations:** 1 Department of Radiation Oncology, Heavy Ion Therapy Research Institute of Yonsei Cancer Center, Yonsei University College of Medicine, Seoul, Republic of Korea; 2 Department of Medical Device Engineering and Management, College of Medicine, Yonsei University, Seoul, Republic of Korea; 3 Department of Biostatistics and Data Science, University of Texas Medical Branch, Galveston, TX, United States

**Keywords:** carbon ion radiotherapy, dose–volume histograms, ProKnow Scoring, prostate cancer, radiation therapy plans, rectal toxicity, robust evaluation, SpaceOAR

## Abstract

**Background:**

Carbon ion radiotherapy (CIRT) offers superior dose conformity and biological effectiveness, making it an effective modality for treating prostate cancer. However, dose escalation is limited by the anatomical proximity of the rectum. Although the SpaceOAR™ hydrogel spacer has demonstrated efficacy in photon-based radiotherapy by reducing rectal dose, its application in CIRT remains insufficiently studied. This study aimed to assess the impact of SpaceOAR on dose distribution and plan robustness in patients with prostate cancer receiving CIRT.

**Methods:**

Twenty-five patients with clinically localized prostate cancer who received SpaceOAR insertion between the prostate and rectum were included. Treatment plans were generated using the RayStation treatment planning system and compared across conventional, rectum-protection, and post-SpaceOAR insertion plans in terms of dosimetry, including dose–volume histograms (DVHs). Robustness evaluation assessed plan stability against patient setup uncertainties, and ProKnow scoring was used to evaluate treatment plan quality.

**Results:**

Compared with conventional CIRT and rectum-protection plans without SpaceOAR, plans with SpaceOAR insertion resulted in a substantial reduction in rectal radiation dose across all 25 patients (p < 0.001). SpaceOAR insertion also improved overall plan quality, as reflected in DVH metrics and ProKnow scores (p < 0.001). Robust evaluation confirmed that SpaceOAR enhanced the safety and reliability of the treatment plans.

**Conclusions:**

Integrating SpaceOAR into CIRT for prostate cancer appears to reduce rectal radiation exposure, improves plan robustness, and enables potential dose escalation to the target volume without compromising safety.

## 1 Introduction

Carbon ion radiotherapy (CIRT), characterized by its distinct Bragg Peak, spares doses to organs at risk (OARs) and provides superior dose distribution compared with photon-based radiotherapy (1, 2). In addition, CIRT exhibits greater biological effectiveness—expressed through linear energy transfer and relative biological effectiveness (RBE)—than proton radiotherapy, the most widely used form of particle therapy. These physical and biological advantages make CIRT well-suited for hypofractionated treatment schedules, allowing effective tumor control with fewer fractions while minimizing normal tissue toxicity (3). Such factors are routinely incorporated into dose calculation and treatment planning to account for the biological effectiveness of CIRT (4–8). Owing to these advantages, CIRT is regarded as an appropriate modality for treating radioresistant tumors while limiting dose exposure to surrounding normal tissues (2, 4, 9–12).

In prostate cancer radiotherapy, rectal toxicity is a major concern owing to the close anatomical relationship between the prostate and rectum, especially when escalating the dose to the target volume. This risk affects the safety of CIRT for prostate cancer. To mitigate this issue, perirectal spacers have been developed to increase the distance between the prostate and rectum, thereby reducing radiation exposure to the rectum (13). Several injectable spacer materials—such as hyaluronic acid, collagen, and polyethylene glycol hydrogels—have shown promising outcomes in clinical studies. Among these, the SpaceOAR™ (Boston Scientific, Marlborough, MA, USA) is one of the most extensively studied. Once injected between the prostate and rectum, the SpaceOAR hydrogel expands and solidifies into a soft, absorbable spacer, effectively increasing separation between the organs. Clinical studies indicate that SpaceOAR is easy to apply, well-tolerated, and significantly reduces rectal radiation dose, leading to improved clinical outcomes (14, 15).

Despite the dosimetric benefits of perirectal spacers such as SpaceOAR, their combination with CIRT has not been extensively investigated (16). This gap highlights the need for further studies to explore the synergistic effects of integrating these two advanced therapies. This study aimed to address this gap by conducting a comparative analysis of CIRT treatment plans for prostate cancer and evaluating plans before and after SpaceOAR insertion using computed tomography (CT). The primary objective was to assess how SpaceOAR affects dose distribution to the prostate and surrounding organs. By comparing pre- and post-SpaceOAR treatment plans, the study aims to quantify dosimetric changes and to evaluate the impact of SpaceOAR on plan robustness and overall planning performance in carbon ion radiotherapy for prostate cancer (17–19).

## 2 Methods and materials

### 2.1 Ethics approval and consent

This study was approved by the Institutional Review Board (IRB) of Severance Hospital and conducted in accordance with the Declaration of Helsinki. The requirement for written informed consent was waived owing to the retrospective nature of the study. Project number: 4-2024-0862.

### 2.2 SpaceOAR

This study included 25 patients with clinically localized prostate cancer who were treated with definitive radiotherapy. Each patient had a SpaceOAR hydrogel inserted percutaneously between the prostate and rectum, positioned within Denonvilliers’ fascia anterior to the rectum. SpaceOAR gel injection was administered prior to CT and MRI simulations.

### 2.3 Patient cohort

From May 2023 to April 2024, we retrospectively identified a representative cohort of 25 patients with prostate cancer who consecutively received CIRT at our institution and met the predefined eligibility criteria (2). All cases involved primary tumors, and all patients received definitive radiotherapy.

All patients underwent CT and magnetic resonance imaging (MRI) for treatment planning after SpaceOAR hydrogel injection. As part of a phase 2 study, patients received hypofractionated radiotherapy (51.60 Gy delivered in 12 fractions of 4.3 Gy) based on the D’Amico classification and were treated with CIRT for low-, intermediate-, and high-risk prostate cancer.

SpaceOAR (10 mL) was systematically injected between the rectum and prostate under local anesthesia and ultrasound guidance. Because the prostate and SpaceOAR were better visualized on T2-weighted MRI than on CT, fusion imaging was subsequently used for target and SpaceOAR delineation.

Eligible patients had T1–T3 stage prostate cancer, Gleason scores ≤ 8, prostate-specific antigen (PSA) levels ≤ 20 ng/mL, and an Eastern Cooperative Oncology Group (ECOG) performance status of 0–1, and were scheduled to receive CIRT.

Exclusion criteria included prostate volume > 80 cm³, metastatic disease, current or recent androgen deprivation therapy, and previous prostate surgery or radiotherapy. Additionally, cases in which the interval between CT scans before and after SpaceOAR insertion exceeded 6 months or in which the difference in prostate volume before and after insertion exceeded 15% were excluded. These criteria were applied because intervals longer than 6 months may result in substantial target changes between pre- and post-SpaceOAR plans, justifying the time limit. Even within 6 months, prostate volume differences greater than 15% could significantly influence the dose impact on surrounding organs during planning.

### 2.4 Carbon planning and details

Fused CT–MRI images were used for target and organ-at-risk delineation to improve visualization of the prostate–rectum interface and hydrogel spacer. Contouring was performed using an institutional workflow informed by the ESTRO ACROP consensus guideline for CT- and MRI-based target volume delineation for primary radiation therapy of localized prostate cancer, with final contouring decisions made by experienced radiation oncologists based on clinical judgment (20). Treatment plans were retrospectively generated using the RayStation treatment planning system (TPS) (version 11B; RaySearch Laboratories, Sweden) for a cohort of 25 patients with prostate cancer (#1–25) undergoing CIRT, using an inverse planning workflow for scanned carbon-ion pencil beam therapy. To evaluate the dosimetric impact of CIRT, patients with and without SpaceOAR insertion were included.

Three distinct treatment plans were developed:

1. A plan prioritizing target coverage using pre-SpaceOAR CT scans.
2. A plan prioritizing rectal dose reduction to predefined thresholds using pre-SpaceOAR CT scans, with these thresholds corresponding to the clinical goals of our institution.
3. A plan using post-SpaceOAR CT scans to simultaneously optimize target coverage and rectal sparing.

**Figure 1:**
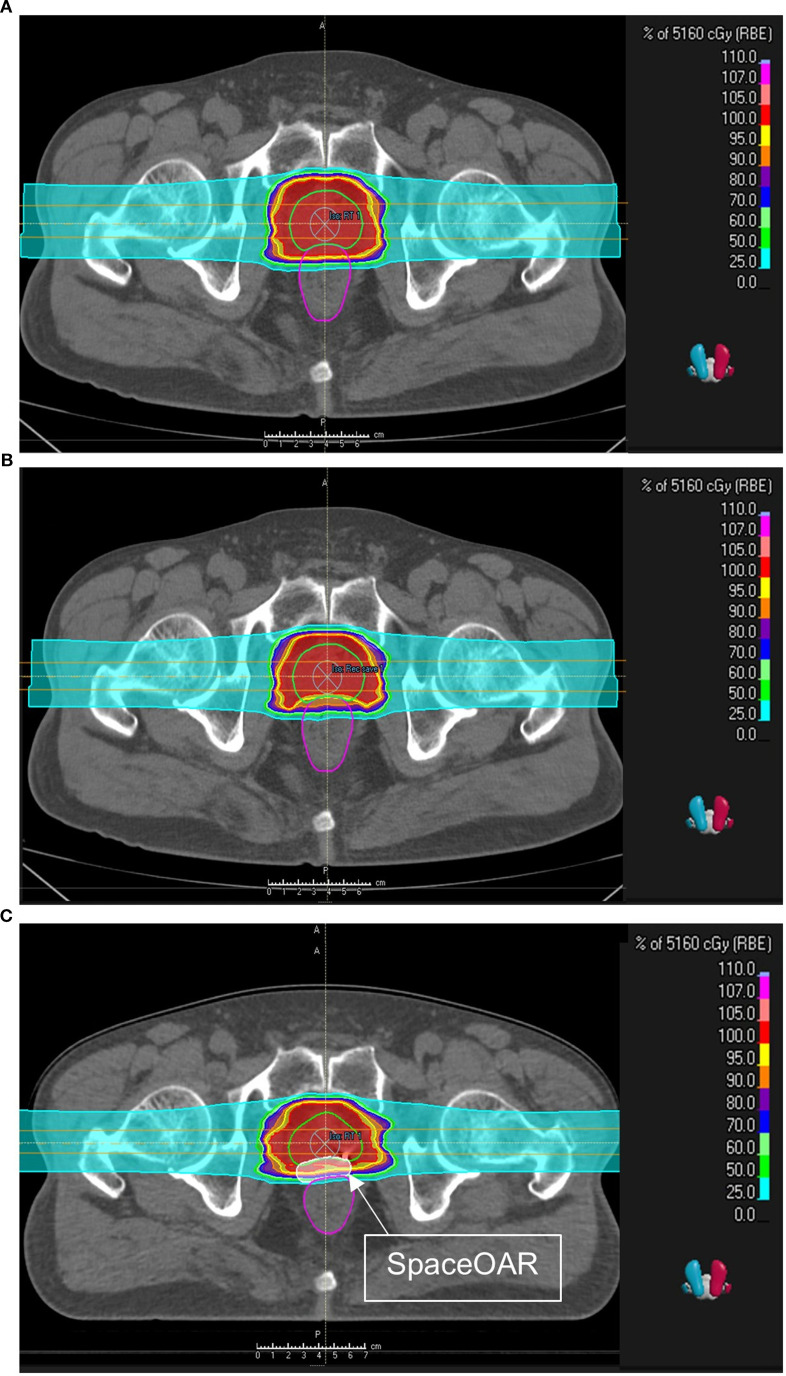
Representative dose distribution of CIRT plans in transverse CT views. **(A)** Pre-SpaceOAR standard plan. **(B)** Pre-SpaceOAR rectum protection plan. **(C)** SpaceOAR insertion plan. The color wash represents dose levels (cGy), highlighting differences in rectal sparing and target coverage across the three planning approaches. These images illustrate the improved rectal sparing achieved with SpaceOAR insertion without compromising target coverage.

The dose constraints were based on our institutional clinical protocol for prostate CIRT, with rectal dose limits designed to reflect dose-limiting concepts reported in Japanese prostate CIRT clinical series. In particular, rectal thresholds were implemented using volume-based limits at representative dose levels (e.g., 95%, 80%, and 50% of the prescription), reflecting commonly reported rectal constraints such as V80% limits, to minimize the risk of late rectal toxicity (21). The total prescribed dose to the planning target volume (PTV) was 51.6 Gy, delivered in 12 fractions, with each of the RT and LT fields delivering 4.3 Gy across six sessions. In all plans, at least 95% of the PTV was covered by the 100% isodose line. The maximum dose did not exceed 107% of the prescribed dose. **Figure 1** illustrates the representative treatment plans generated using RayStation, enabling direct comparison of beam arrangements among the pre-SpaceOAR, pre-SpaceOAR with rectal protection, and post-SpaceOAR (**Figures 1A–C**).

### 2.5 Evaluations

#### 2.5.1 Assessment of patient characteristics

Baseline clinical parameters were obtained from electronic medical records before the initiation of CIRT. Tumor staging was determined based on clinical examination and imaging findings. ECOG performance status was assessed at the time of treatment planning. Gleason scores were determined from prostate biopsy specimens, and initial prostate-specific antigen (iPSA) levels were measured using pretreatment blood tests. Based on these parameters, patients were classified into low-, intermediate-, and high-risk groups according to the standard National Comprehensive Cancer Network risk classification, which incorporates clinical T stage, Gleason score, and iPSA levels.

#### 2.5.2 Treatment planning system

Plan evaluations were conducted using the dose distributions generated by the TPS, the clinical goals specified for each patient, and dose–volume histogram (DVH) values for relevant organs. DVHs for the prostate (PTV), rectum, and penile bulbs were calculated for all patients. For the PTV, the minimum (Dmin), maximum (Dmax), and mean doses (Dmean) were evaluated. However, owing to substantial differences in bladder volume before and after SpaceOAR insertion, DVH values for the bladder were not evaluated in the TPS, as volume variability could confound the interpretation of percentage-based DVH metrics.

The clinical effectiveness of mMKM without further modification has been validated at CIRT centers in Japan (2, 22–24).

#### 2.5.3 Prostate–rectum separation and rectal DVH analysis

**Figure 2:**
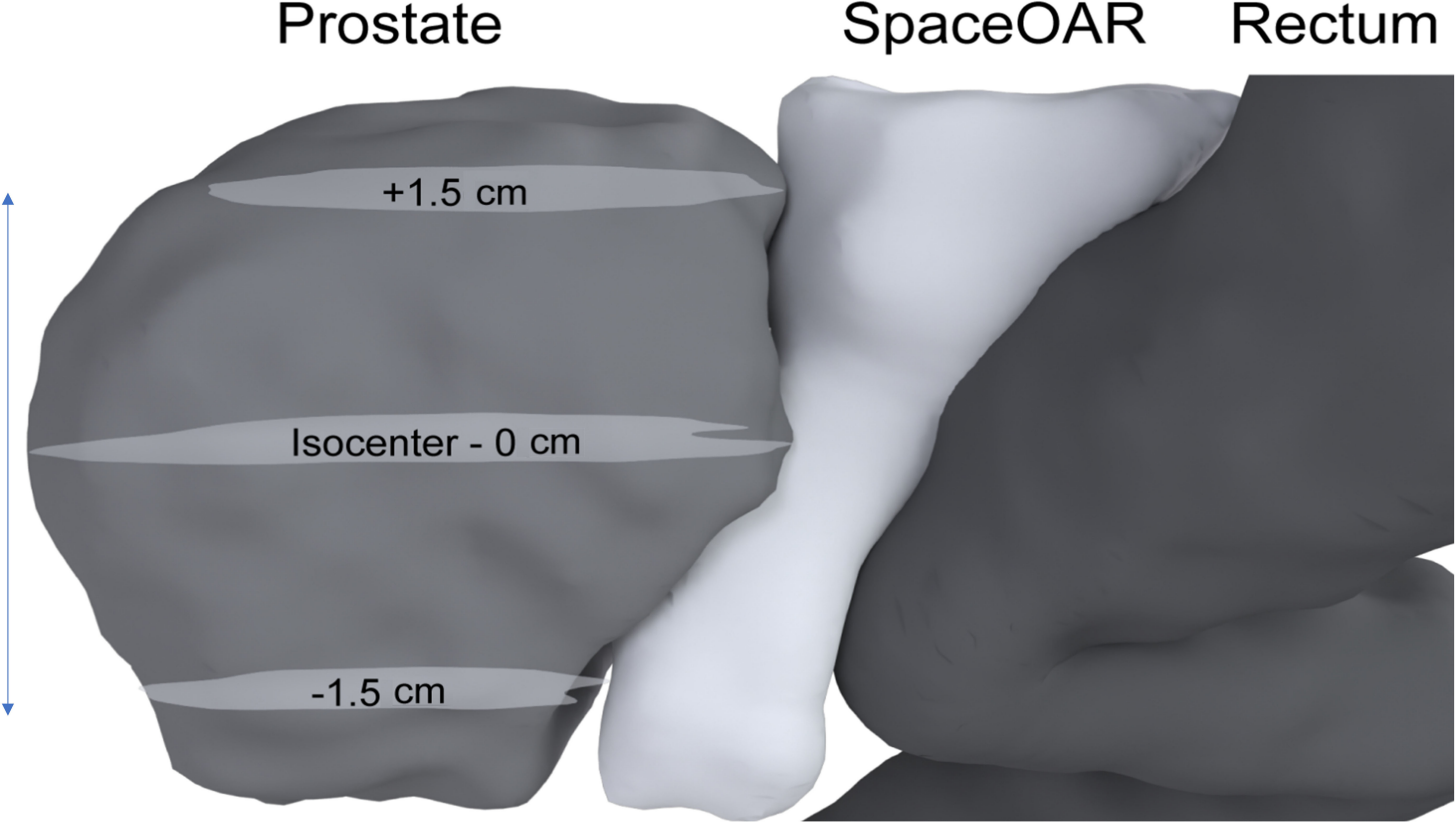
Illustration of different prostate levels in the sagittal plane. The isocenter at 0 cm represents the center of the prostate. The +1.5 cm point is located 1.5 cm superior to the 0 cm, while the -1.5 cm point is 1.5 cm inferior to the 0 cm.

**Figure 3:**
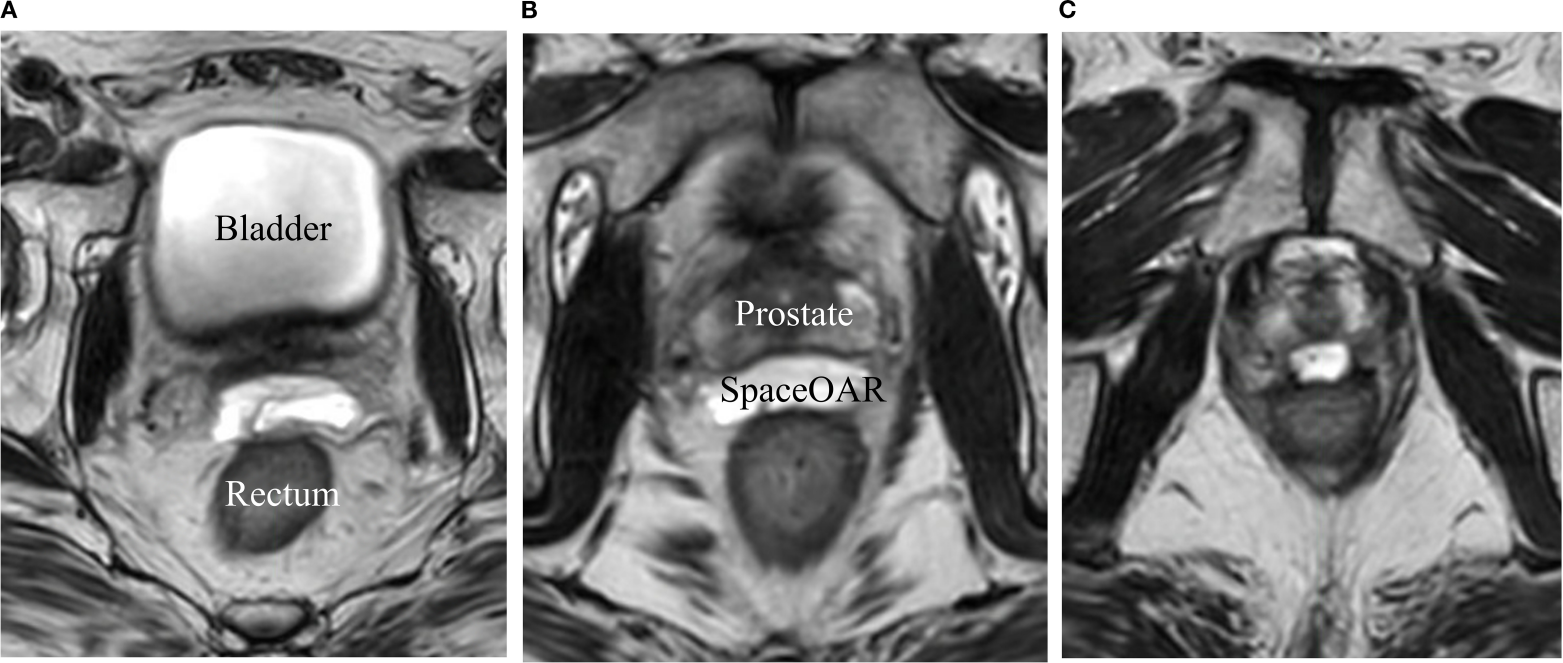
Transverse view of the T2 magnetic resonance image (T2-MRI). **(A)** +1.5 cm from the center of the prostate, **(B)** Center of the prostate, **(C)** -1.5 cm from the center of the prostate.

To evaluate prostate–rectum separation before and after SpaceOAR insertion, patients were categorized according to the measured distance between the prostate and rectum. CT–MRI fusion was used to enhance measurement accuracy. For each patient, the separation distance was calculated by averaging the distances obtained at three central prostate reference points: 0 cm, +1.5 cm (superior), and −1.5 cm (inferior) (**Figures 2**, **3A–C**). Based on these values, patients were classified into four groups: 0–5 mm, 5–10 mm, 10–15 mm, and >15 mm. For all 25 patients, the mean and standard deviation (SD) at each reference position (0 cm, +1.5 cm, −1.5 cm) were calculated from the images acquired before and after SpaceOAR insertion.

The anatomical cross-section and approximate dose distribution during SpaceOAR insertion between the prostate and rectum under ultrasound guidance are shown in **Supplementary Figure 1**.

For each patient, DVH values for the rectum were calculated using the average prostate–rectum separation distances measured at 0 cm, +1.5 cm, and −1.5 cm reference positions. This analysis aimed to evaluate the distribution of radiation dose to the rectum based on the degree of separation. The DVH parameters analyzed included Dmin, Dmax, Dmean, V15%, V20%, V30%, V40%, and V50%. These data were used to assess changes in rectal radiation dose following SpaceOAR insertion. Using this approach, the impact of SpaceOAR insertion on rectal sparing during CIRT in patients with prostate cancer was quantitatively analyzed.

#### 2.5.4 Robust evaluation of treatment plans

A robust evaluation passing rate is a concept in radiation therapy that ensures the effectiveness and safety of treatment plans by accounting for various uncertainties.

**Figure 4:**
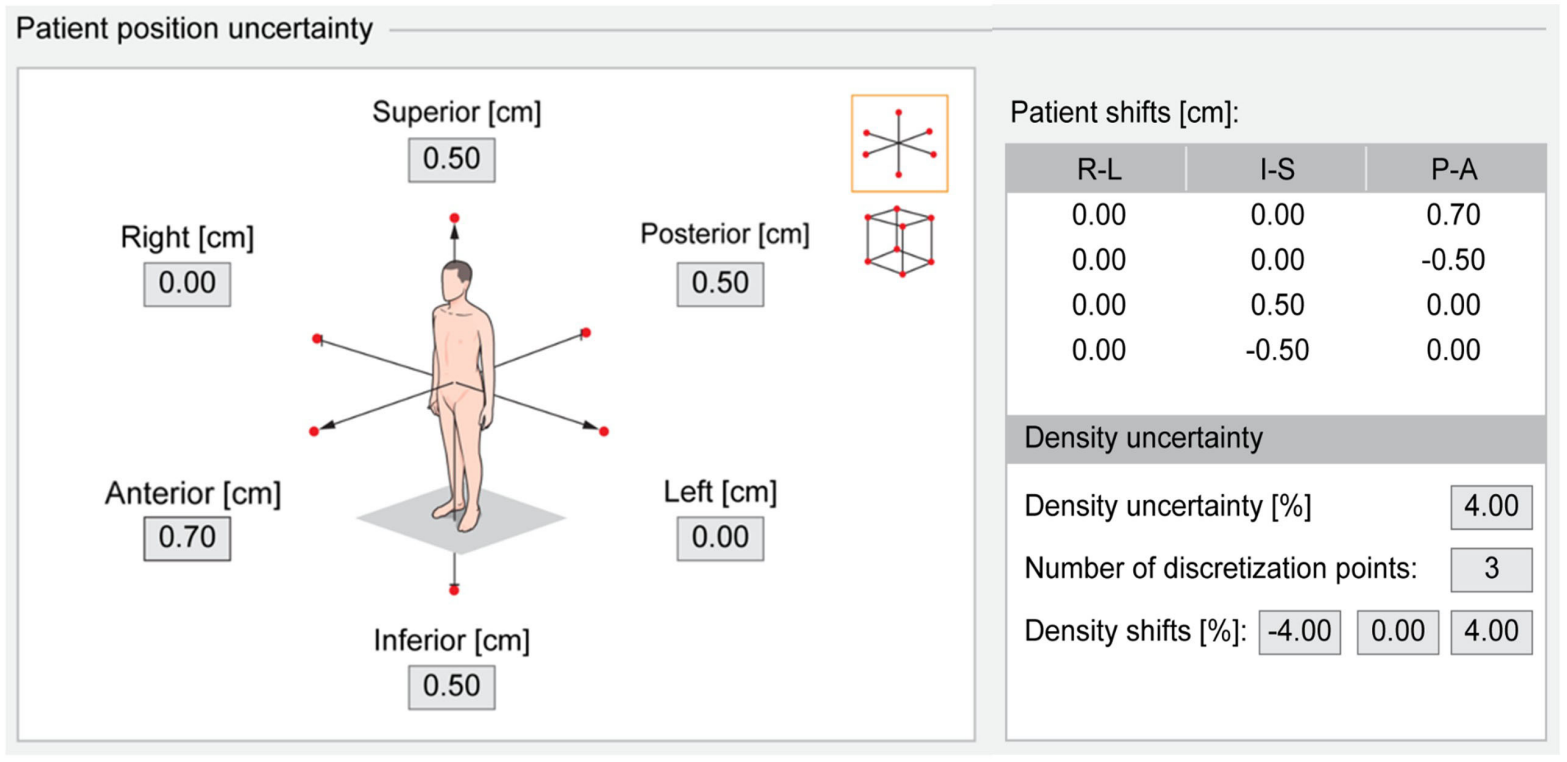
Robust evaluation of patient position and density uncertainty in RayStation.

Patient position uncertainty was modeled as anisotropic shifts in six directions, with movements up to ±0.50 cm in certain directions and ±0.70 cm in others. Specifically, uncertainties in the superior, inferior, and posterior directions were modeled at ±0.50 cm, whereas the anterior direction, where the prostate and rectum are in close proximity, was evaluated more stringently at ±0.70 cm. Additionally, density uncertainty was modeled by scaling the mass density of the patient by ±4% using three discretization points. These modeling approaches, combined with the planning criteria detailed in **Table 1**, ensure treatment plan robustness by accounting for various patient-positioning scenarios and density variations (25) (**Figure 4**).

**Table 1:** Dosimetric criteria for treatment plans.

Organ	Dosimetric criteria
Prostate	At least 49.02 Gy (RBE) covering 99.9% of the volume
	At least 49.02 Gy (RBE) covering 100.0% of the volume
Rectum	Maximum 1.00 cm³ volume at 49.02 Gy (RBE)
	Maximum 5.00 cm³ volume at 41.28 Gy (RBE)
	Maximum 10.00 cm³ volume at 25.80 Gy (RBE)
Bladder	Volume receiving 44.89 Gy (RBE) < 25%
	Volume receiving 28.38 Gy (RBE) < 45%
Penile bulb	Dose to a 0.03 cm³ volume

Anatomical sites, such as the prostate, rectum, and bladder, were included in the evaluation. The passing rates for these organs were assessed based on the criteria listed in **Table 1**. The mean and SD of the passing rates were calculated for the three conditions: pre-SpaceOAR, pre-SpaceOAR with rectal protection, and post-SpaceOAR.

#### 2.5.5 ProKnow scoring

ProKnow^®^ (ProKnow, LLC) scoring was utilized to evaluate the quality of treatment plans for patients with prostate cancer undergoing CIRT. This method involves analyzing specific metrics for the prostate, rectum, bladder, and penile bulb, with each metric assigned an objective based on clinical goals. Scores were calculated to reflect treatment plan compliance with these goals, ranging from “Ideal” to “Unacceptable.” The criteria and their respective weights were determined by two radiation oncologists to ensure comprehensive evaluation. Composite scores were generated by combining individual metric scores, allowing for comparisons across treatment plans (26) (**Tables 1** and **2**).

**Table 2:** Plan metrics collected for each prostate CIRT plan.

No	Metric description	Minimum requirement	Ideal	Interval	Weight
1	Volume (%) of bladder covered by 44.89 (Gy)	10	25	5	1
2	Volume (%) of bladder covered by 28.38 (Gy)	15	45	10	1
3	Dose (Gy) covering 99.90 (%) of the prostate	49.02	51.6	PTV coverage 1–2% difference	1
4	Dose (Gy) covering 100.00 (%) of the prostate	49.02	51.6	PTV coverage 1–2% difference	1
5	Volume (cc) of the rectum covered by 49.02 (Gy)	0.4	1	0.2	2
6	Volume (cc) of the rectum covered by 41.28 (Gy)	2	5	1	2
7	Volume (cc) of the rectum covered by 25.80 (Gy)	1	10	3	2
8	Dose (Gy) covering 0.03 (cc) of the penile bulb	20	50	10	1

A total of eight evaluation metrics were applied, resulting in a maximum composite score of 64 points.

### 2.6 Statistical analysis

Continuous variables (age, iPSA) were summarized as mean, SD, median, minimum (min), and maximum (max), whereas categorical variables (ECOG performance status, T stage, and Gleason score) were summarized as frequencies and proportions. Descriptive analyses were performed for the entire sample and separately for each risk group (low, intermediate, and high). Additionally, the dose distribution indices, prostate–rectum distances, DVH values, robustness evaluation pass rates, and total ProKnow scores for each plan were summarized using the mean and SD.

A one-way repeated measures analysis of variance (ANOVA) was conducted to determine whether the DVH results for the prostate, rectum, and penile bulb differed significantly across the three plans (1. Pre-SpaceOAR, 2. pre-SpaceOAR with rectal protection, 3. Post-SpaceOAR). If significant differences were detected, the Bonferroni correction was applied to identify specific plan differences. ProKnow scores were also compared across the three plans to assess the differences in overall plan quality.

Two-way repeated measures ANOVA was used to evaluate the effect of SpaceOAR (first factor) and three anatomical positions from the prostate center (+1.5 cm, 0 cm, −1.5 cm; second factor) on the prostate–rectum distance. Rectal robustness evaluation was analyzed using the three treatment plans (Pre-SpaceOAR, Pre-SpaceOAR with rectal protection, Post-SpaceOAR) and pass rates (%) for three volume thresholds (Max 1 cm³, Max 5 cm³, Max 10 cm³). The Bonferroni correction was applied in cases of significant interaction effects.

Finally, regression analyses were performed to assess the relationship between rectal DVH values (Dmin, Dmax, Dmean, V15, V20, V40, and V50) and the degree of prostate–rectum separation. Beta regression with a logistic link function was used for outcomes expressed as percentages (V15, V20, V40, and V50), and ordinary least squares regression was applied for Dmin, Dmax, and Dmean.

All statistical analyses were conducted using R Statistical Software (version 4.1.3; R Foundation for Statistical Computing, Vienna, Austria), with the ‘mgcv’ package used for beta regression modeling. Statistical significance was set at p < 0.05.

## 3 Results

### 3.1 Patient characteristics

Twenty-five patients with prostate cancer were included in this study and classified into low-risk (N = 6), intermediate-risk (N = 16), and high-risk (N = 3) groups. The demographic characteristics of the patients are as follows: the mean age at the time of radiotherapy was 68.96 years (SD 7.86), with 65.00 years (SD 5.76) for the low-risk group, 67.81 years (SD 6.34) for the intermediate-risk group, and 83.00 years (SD 2.00) for the high-risk group. ECOG performance status PS0 and PS1 were noted for all patients, with no patients classified as PS2 or higher. T stages ranged from T1b to T3a, and Gleason scores of 6, 7, and 8 were observed. iPSA levels were higher in the high-risk group, with a median iPSA of 8.93 ng/mL (range: 7.69–14.00) compared to median values of 5.10 ng/mL and 5.44 ng/mL in the low- and intermediate-risk groups, respectively (**Table 3**).

**Table 3:** Demographic and baseline characteristics data.

Characteristic	Low risk group	Intermediate risk group	High risk group	Total
	(N = 6)	(N = 16)	(N = 3)	(N = 25)
Age (years)				
N	6	16	3	25
Mean (SD)	65.00 (5.76)	67.81 (6.34)	83.00 (2.00)	68.96 (7.86)
Median	67	67	83	68
Min, Max	57, 70	58, 78	81, 85	57, 85
ECOG performance status, N (%)				
PS0	2 (33.33)	2 (12.50)	1 (33.33)	5 (20.00)
PS1	4 (66.67)	14 (87.50)	2 (66.67)	20 (80.00)
PS2	0 (0.00)	0 (0.00)	0 (0.00)	0 (0.00)
PS3	0 (0.00)	0 (0.00)	0 (0.00)	0 (0.00)
PS4	0 (0.00)	0 (0.00)	0 (0.00)	0 (0.00)
T stage, N (%)				
T1b	1 (16.67)	0 (0.00)	0 (0.00)	1 (4.00)
T1c	2 (33.33)	0 (0.00)	0 (0.00)	2 (8.00)
T2a	3 (50.00)	7 (43.75)	1 (33.33)	11 (44.00)
T2b	0 (0.00)	3 (18.75)	0 (0.00)	3 (12.00)
T2c	0 (0.00)	6 (37.50)	0 (50.00)	6 (24.00)
T3a	0 (0.00)	0 (0.00)	2 (66.67)	2 (8.00)
T3b	0 (0.00)	0 (0.00)	0 (00.00)	0 (0.00)
Gleason Score (%)				
6	6 (100.00)	3 (13.33)	0 (00.00)	9 (36.00)
7	0 (0.00)	13 (86.67)	1 (33.33)	14 (56.00)
8	0 (0.00)	0 (0.00)	1 (33.33)	1 (4.00)
(-)	0 (0.00)	0 (0.00)	1 (33.33)	1 (4.00)
Initial PSA (iPSA)				
N	6	15	4	25
Mean (SD)	4.95 (0.84)	5.57 (2.07)	10.21 (3.34)	5.98 (2.47)
Median	5.10	5.44	8.93	5.30
Min, Max	3.56, 6.06	2.77, 10.80	7.69, 14.00	2.77, 14.00

N(%): The number and ratio of participants were calculated for each group.

(-): It indicates that the Gleason Score was not assessed.

### 3.2 Comparison of dosimetry indices

The contours of the prostate, rectum, bladder, and penile bulb were delineated for all patients. The volumes of these organs are presented in **Supplementary Table 1**. These contours were used to generate dose statistics for each patient across the three treatment plans: pre-SpaceOAR, pre-SpaceOAR with rectal protection, and post-SpaceOAR. The values of Dmin, Dmax, Dmean, and V15–V50 are presented as means with SDs in **Table 4**, and the corresponding p-values were obtained from statistical comparisons among the three treatment plans across all 25 patients.

**Table 4:** Mean DVH Values Pre-SpaceOAR, Pre-SpaceOAR with Rectum Protection, and Post-SpaceOAR.

		Prostate			Rectum											Penile bulb										
Treatment plan	Statistic	Dmin (Gy)	Dmax (Gy)	Dmean (Gy)	Dmin (Gy)	Dmax (Gy)	Dmean (Gy)	V_15_ (%)	V_20_ (%)	V_25_ (%)	V_30_ (%)	V_35_ (%)	V_40_ (%)	V_45_ (%)	V_50_ (%)	Dmin (Gy)	Dmax (Gy)	Dmean (Gy)	V_15_ (%)	V_20_ (%)	V_25_ (%)	V_30_ (%)	V_35_ (%)	V_40_ (%)	V_45_ (%)	V_50_ (%)
Pre-Space OAR	Mean	51.46	52.30	51.91	0.08	52.83	9.85	21.60	19.17	17.32	15.67	13.98	12.24	10.61	7.85	2.88	51.31	16.06	37.73	29.93	28.86	27.28	20.46	19.35	18.08	11.12
	SD*	0.12	0.47	0.11	0.03	0.39	1.77	4.09	3.70	3.31	2.95	2.72	2.37	2.03	1.61	11.03	7.08	7.02	16.81	13.93	13.84	13.26	10.91	10.10	9.58	7.08
Pre-Space OAR (Rectum Protection)	Mean	46.54	53.86	52.67	0.08	50.81	8.58	20.27	17.66	15.52	13.49	11.35	9.12	6.35	0.06	0.46	52.64	19.72	45.28	37.97	35.84	33.81	28.23	24.97	23.19	15.52
	SD*	3.58	2.70	1.56	0.03	0.82	1.80	4.54	3.92	3.43	3.21	2.68	2.77	2.28	0.06	0.20	1.04	7.32	16.61	15.74	14.28	13.95	13.40	11.75	11.29	9.23
	*p*-value^1^	< 0.001	0.025	0.071	0.013	< 0.001	< 0.001	0.015	0.006	0.003	0.001	< 0.001	< 0.001	< 0.001	< 0.001	< 0.001	0.060	< 0.001	< 0.001	< 0.001	< 0.001	< 0.001	< 0.001	< 0.001	< 0.001	< 0.001
Post-Space OAR	Mean	51.47	52.26	51.90	0.09	50.66	3.32	6.97	5.31	4.34	3.57	2.67	1.97	1.46	0.67	0.41	53.73	16.98	37.38	33.65	29.50	25.41	23.16	20.01	15.93	12.50
	SD*	0.09	0.07	0.02	0.03	4.70	1.88	4.65	3.84	3.50	3.25	2.65	2.22	1.91	1.12	0.18	2.91	6.82	16.26	14.12	13.66	13.11	11.49	10.59	10.27	8.45
	*p*-value^2^	> 0.999	> 0.999	> 0.999	0.339	0.086	< 0.001	< 0.001	< 0.001	< 0.001	< 0.001	< 0.001	< 0.001	< 0.001	< 0.001	0.981	0.411	< 0.001	< 0.001	< 0.001	< 0.001	< 0.001	< 0.001	< 0.001	< 0.001	< 0.001

1 Pre-SpaceOAR VS Pre-SpaceOAR (Rectum Protection).

2 Pre-SpaceOAR VS Post-SpaceOAR.

For the prostate, significant differences were found in Dmin (p < 0.001) and Dmax (p = 0.025) between pre-SpaceOAR and pre-SpaceOAR with rectal protection; however, no significant difference was observed in Dmean (p = 0.071). There were no significant differences between pre- and post-SpaceOAR in any of the metrics (p > 0.999).

For the rectum, significant differences were observed in Dmin (p = 0.013) between pre-SpaceOAR and pre-SpaceOAR with rectal protection, whereas no significant differences were found between pre-SpaceOAR and post-SpaceOAR (p = 0.339). For Dmax, the p-values were <0.001 and 0.086, respectively, and all comparisons of Dmean showed p-values <0.001. Additionally, significant reductions in rectal volume percentages (V15–V50) were observed, with all p-values <0.05.

For the penile bulb, significant differences were found in Dmin (p < 0.001) and Dmean (p < 0.001) between pre-SpaceOAR and pre-SpaceOAR with rectal protection; however, no significant difference was observed in Dmax (p = 0.060). Comparing pre-SpaceOAR and post-SpaceOAR, there were no significant differences in Dmin (p = 0.981) and Dmax (p = 0.411), but a significant difference in Dmean (p < 0.001). Significant changes in penile bulb volume percentages (V15–V50) were also observed, with all p-values <0.001.

**Figure 5:**
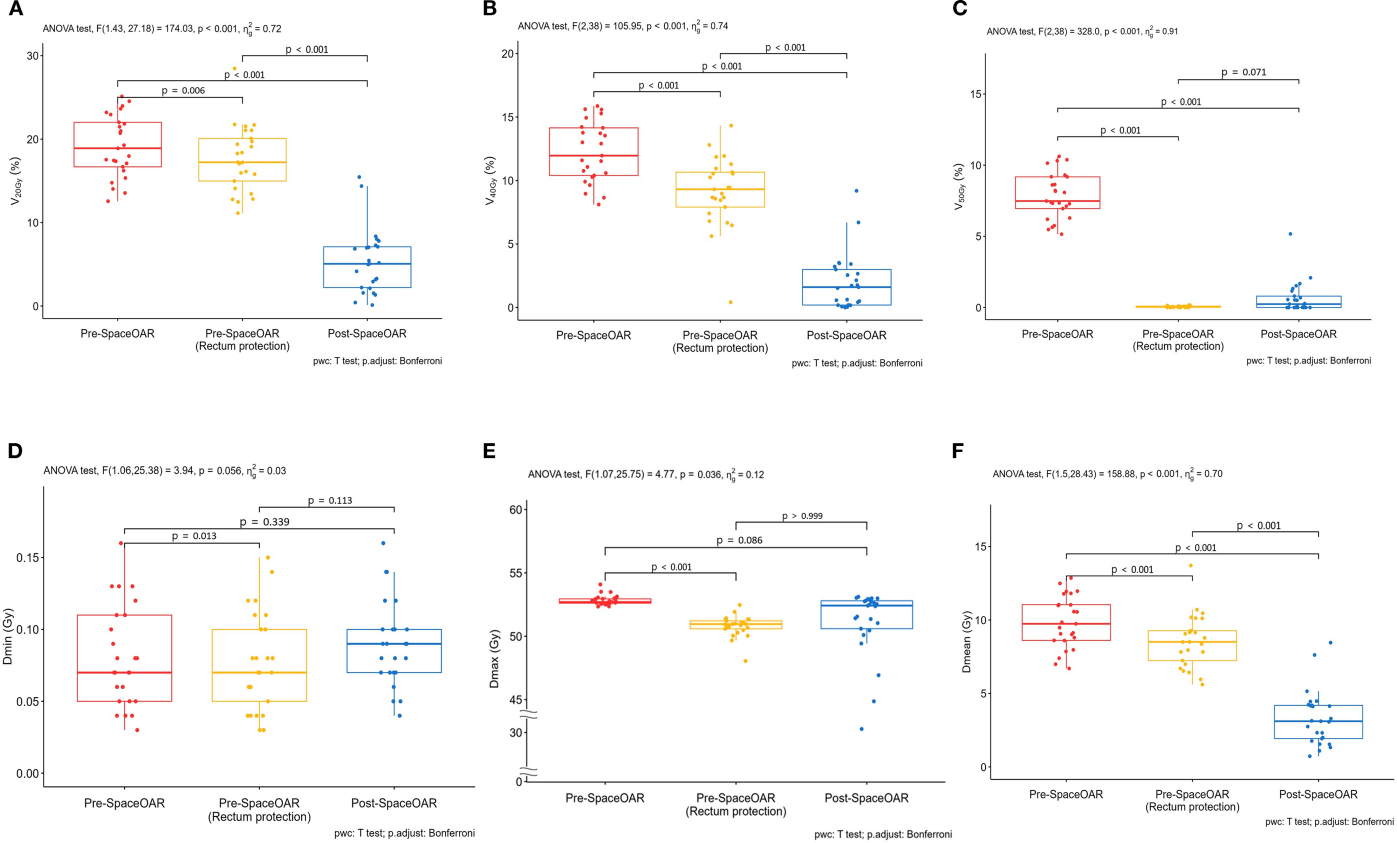
Rectal dosimetric outcomes for Pre-SpaceOAR (Standard Plan), Pre-SpaceOAR (Rectum Protection Plan), and Post-SpaceOAR. **(A)** V20Gy (%), **(B)** V40Gy (%), **(C)** V50Gy (%), **(D)** Dmin (Gy), **(E)** Dmax (Gy), and **(F)** Dmean (Gy).

**Figure 5** illustrates the distribution of rectal DVH parameters across the three treatment plans. Overall reductions in rectal dose metrics were observed following SpaceOAR insertion. In particular, volume-based parameters such as V20 and V40, as well as rectal mean dose, demonstrated statistically significant improvements, whereas V50 and extreme dose metrics (Dmin and Dmax) did not show consistent statistical significance across all plan comparisons.

### 3.3 Prostate–rectum separation results

The separation distance between the prostate and rectum for each patient was calculated by averaging the measurements taken at three reference points: 0 cm (central), +1.5 cm (superior), and −1.5 cm (inferior) (**Figures 2**, **3A–C**). Based on these average distances, the patients were divided into four groups: 0–5 mm (1 patient), 5–10 mm (11 patients), 10–15 mm (11 patients), and >15 mm (2 patients) (**Table 5**).

**Table 5:** Patient cases based on Prostate-Rectum separation.

Prostate–rectum separation	Patient cases
0–5 mm	1
5–10 mm	11
10–15 mm	11
>15 mm	2

Patient cases are organized according to the degree of separation between the prostate and rectum. The degree of separation was determined as the mean of the separation distances measured at the 0 cm, +1.5 cm, and -1.5 cm positions within the prostate.

**Figure 6:**
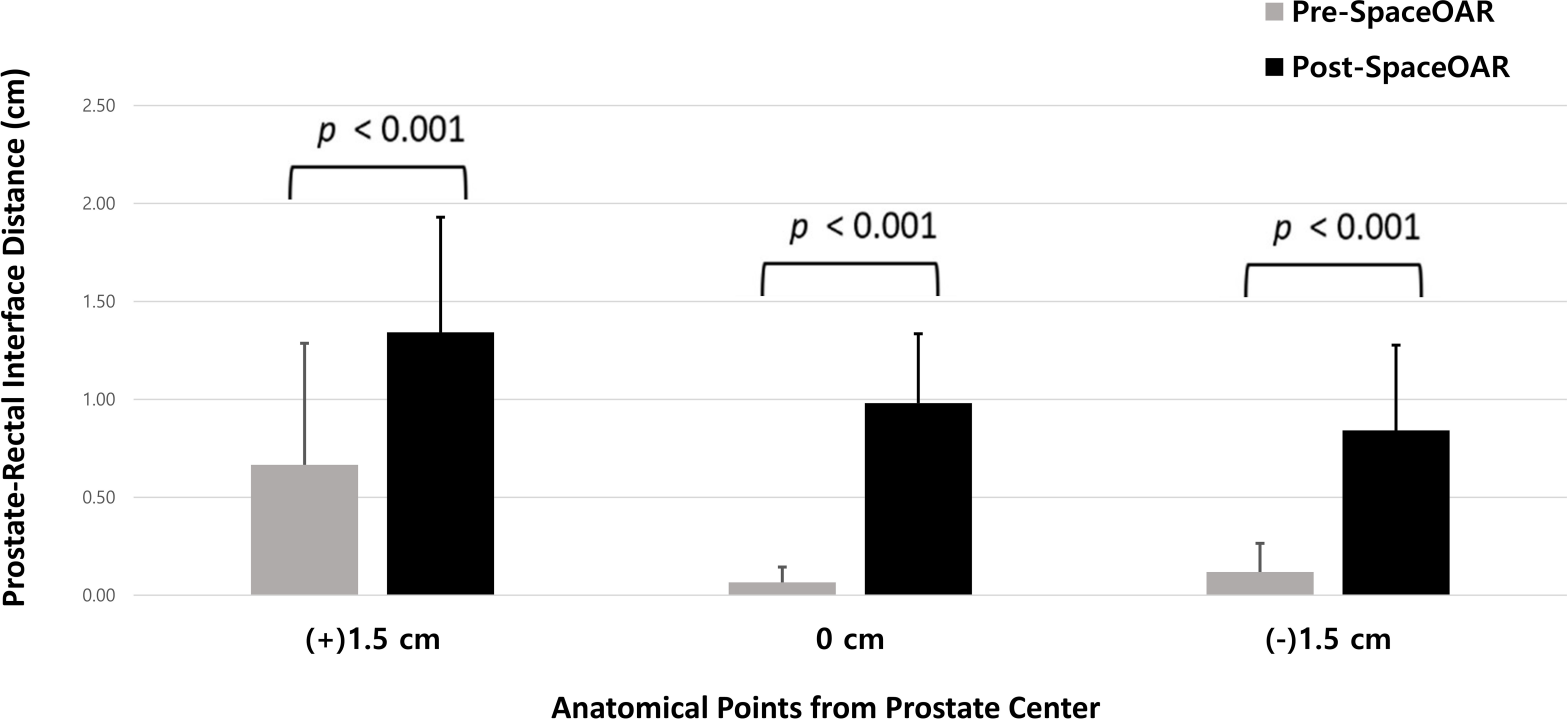
Prostate-rectum separation before and after injection of SpaceOAR at different levels of the prostate.

The introduction of SpaceOAR significantly increased the separation between the prostate and rectum at all measured levels. The mean separation distances and their SD were recorded for both the pre- and post-SpaceOAR conditions. Pre-SpaceOAR measurements showed mean separations of 0.67 cm (SD 0.62) at +1.5 cm, 0.07 cm (SD 0.08) at 0 cm, and 0.12 cm (SD 0.15) at −1.5 cm. Post-SpaceOAR, the separations increased to 1.34 cm (SD 0.59) at +1.5 cm, 0.98 cm (SD 0.35) at 0 cm, and 0.84 cm (SD 0.44) at −1.5 cm. These differences were statistically significant (p < 0.001) at all three measurement points, demonstrating the effectiveness of SpaceOAR in increasing prostate–rectum separation across anatomical levels (**Figure 6**).

### 3.4 Rectum DVH analysis

**Figure 7:**
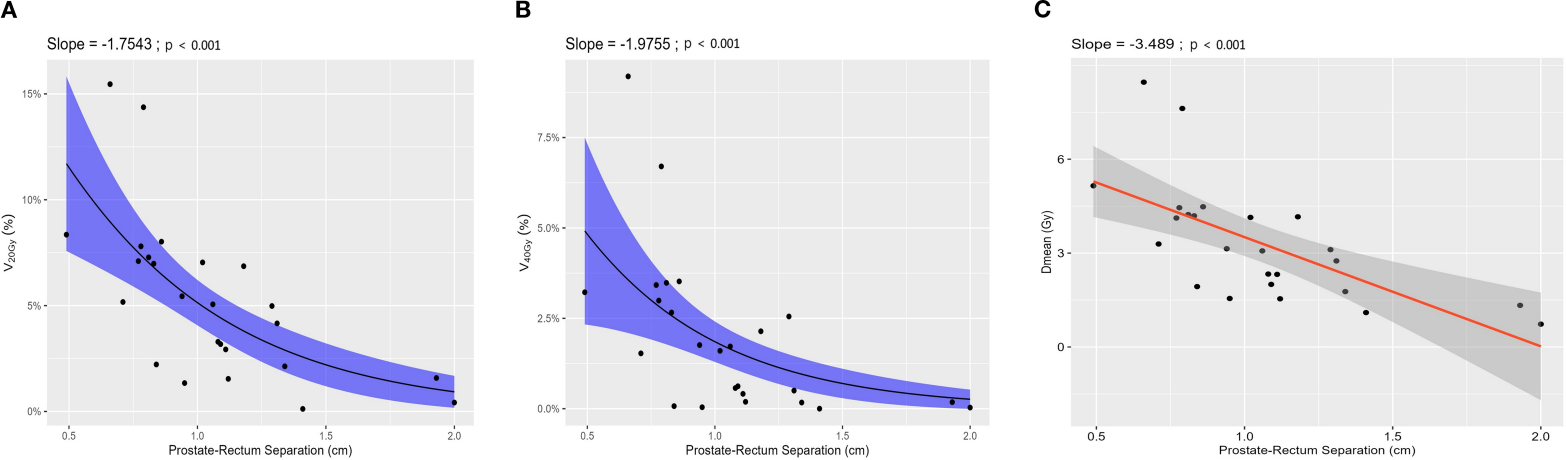
Rectum DVH values by Prostate-Rectum separation degree. **(A)** V20Gy (%), **(B)** V40Gy (%) and **(C)** Dmean (Gy).

Rectal DVH values according to the degree of prostate–rectum separation are summarized in **Supplementary Table 2** and **Figures 7A–C**. **Supplementary Table 2** presents rectal DVH metrics stratified by separation distance, including volume-based parameters and mean dose. Overall, increasing prostate–rectum separation was associated with reduced rectal dose exposure.

**Figure 7** illustrates the relationship between prostate–rectum separation and selected rectal DVH parameters. Volume-based parameters, including V20 and V40, demonstrated significant negative correlations with separation distance (p < 0.05; **Figures 7A,B**), indicating a progressive reduction in the irradiated rectal volume with increasing anatomical separation. In addition, rectal mean dose (Dmean) showed a significant decreasing trend as prostate–rectum separation increased (p < 0.001; **Figure 7C**).

For clarity and conciseness, V20, V40, and Dmean are presented in the main text as representative DVH metrics, whereas the remaining rectal DVH parameters (V15, V50, Dmin, and Dmax) are provided in the **Supplementary Material** (**Supplementary Figure 2**).

### 3.5 Robust evaluation

A robust evaluation was performed to assess plan stability across the three conditions: pre-SpaceOAR, pre-SpaceOAR with rectal protection, and post-SpaceOAR. Passing rates were evaluated based on the dose criteria specified in **Table 1**, with results summarized for the prostate, rectum, and bladder in **Table 6**.

**Table 6:** Robust evaluation passing rates for anatomical organs.

Anatomical Sites	Pre-SpaceOAR		Pre-SpaceOAR (Rectum Protection)		Post-SpaceOAR	
	Mean	SD	Mean	SD	Mean	SD
Prostate	40.06	44.10	0.00	0.00	37.81	39.07
Rectum	40.77	22.58	60.35	11.98	83.33	15.71
Bladder	100.00	0.00	100.00	0.00	100.00	0.00

Mean and standard deviation (SD) values are presented as percentages (%).

For the prostate and bladder, no consistent improvement in robustness was observed across the three planning conditions. In the pre-SpaceOAR rectum-protection plans, the prostate passing rate was 0%, with none of the evaluated scenarios meeting all prostate coverage criteria specified in **Table 1**. In contrast, rectal robustness demonstrated a clear and progressive improvement from pre-SpaceOAR to pre-SpaceOAR with rectal protection, with the highest passing rates observed in post-SpaceOAR plans.

**Figure 8:**
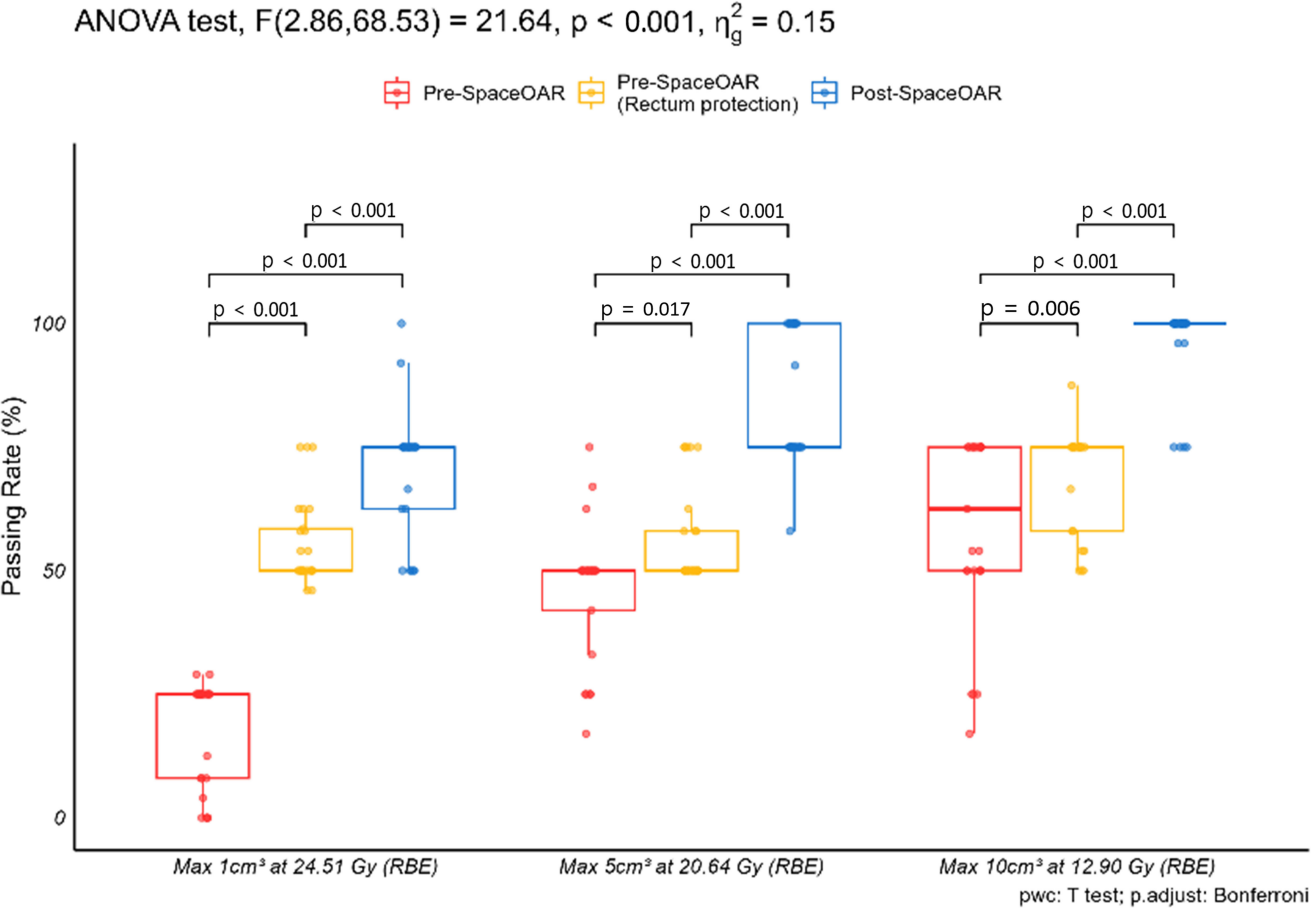
Rectum: Robust evaluation passing rate results by criteria.

Since the rectum is the primary OAR in prostate radiotherapy, subsequent analyses focused on rectal robustness. As shown in **Figure 8**, rectal passing rates were significantly higher in post-SpaceOAR plans compared with both pre-SpaceOAR and pre-SpaceOAR with rectal protection across all evaluated dose criteria (p < 0.05). In addition, pre-SpaceOAR plans with rectal protection showed significantly higher passing rates than pre-SpaceOAR plans without rectal constraints. These findings indicate that SpaceOAR insertion substantially improves the robustness of rectal dose delivery under uncertainty.

### 3.6 ProKnow scoring

ProKnow scoring was performed to compare overall plan quality across three planning conditions: pre-SpaceOAR with standard planning, pre-SpaceOAR with rectal protection planning, and post-SpaceOAR. Composite scores were calculated by aggregating evaluation criteria for the prostate, rectum, bladder, and penile bulb, as defined in **Table 1**.

**Figure 9:**
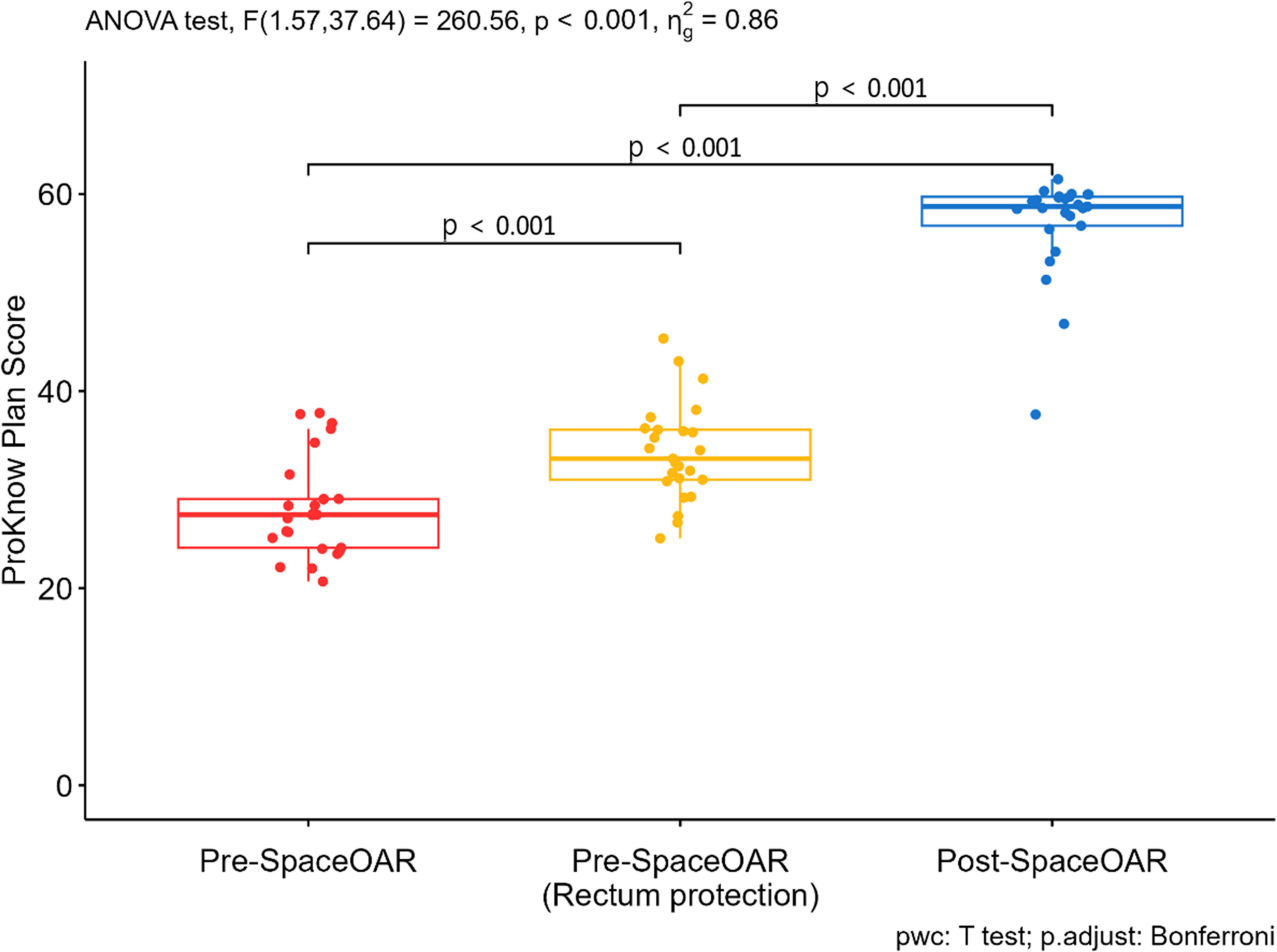
Comparison of p-values from ProKnow plan evaluation.

As summarized in **Supplementary Table 3**, mean ProKnow scores increased sequentially from pre-SpaceOAR to pre-SpaceOAR with rectal protection and were highest in post-SpaceOAR plans. Post-SpaceOAR plans demonstrated a statistically significant improvement in composite scores compared with both pre-SpaceOAR and pre-SpaceOAR with rectal protection plans (p < 0.001; **Figure 9**).

## 4 Discussion

This study evaluated the effect of SpaceOAR insertion on the dosimetric characteristics and planning performance of CIRT plans for prostate cancer. SpaceOAR insertion was associated with significantly reduced rectal dose while preserving prostate target coverage, suggesting a potential improvement in overall plan quality (13, 14). Despite these reductions, prostate dosimetric indices (Dmin, Dmax, and Dmean) did not differ before and after SpaceOAR insertion, indicating effective rectal sparing without compromising therapeutic dose delivery. In the rectum, Dmean significantly decreased following SpaceOAR placement, while Dmin and Dmax remained unchanged, supporting enhanced rectal protection without the need for additional plan modifications (13, 14).

Interestingly, while previous phase III randomized trials using intensity-modulated radiotherapy (IMRT) reported that SpaceOAR significantly decreased the mean dose to the penile bulb and improved long-term erectile function (14), our CIRT dosimetric study demonstrated an opposite trend. In our analysis, the Dmean to the penile bulb inadvertently increased from 16.06 Gy to 16.98 Gy following spacer insertion.

This discrepancy is largely attributable to the distinct beam arrangements utilized in our CIRT workflow. Unlike photon IMRT or VMAT, which utilize multiple beam angles or rotational arcs to sculpt the dose around the penile bulb, our CIRT plans for prostate cancer primarily utilize opposed bilateral beams (left and right lateral beams). When the hydrogel spacer displaces the prostate anteriorly, this spatial shift can unintentionally elevate the penile bulb into the direct trajectory of these lateral carbon-ion beams or move it closer to the sharp high-dose gradient at the prostate apex. Because the radiation dose to the penile bulb is a well-established predictor of radiation-induced erectile dysfunction, this increase warrants clinical attention. However, it is important to emphasize that the absolute Dmean to the penile bulb in our SpaceOAR plans (16.98 Gy) remained strictly within the clinically accepted tolerance limits for preserving erectile function (equivalent Dmean < 50 Gy). Thus, while the relative dose increased, the absolute risk of severe erectile dysfunction is expected to remain low. Nevertheless, our findings highlight a crucial technical caveat: when utilizing a hydrogel spacer with fixed lateral beams in CIRT, radiation oncologists must meticulously evaluate the dose to the penile bulb at the prostate apex, balancing profound rectal sparing against the potential impact on sexual function (**Table 4**) (27).

The pre-SpaceOAR rectum-protection plan, while effective in reducing rectal dose, led to reduced prostate coverage in several cases, resulting in lower overall plan quality. In **Table 6**, the pre-SpaceOAR rectum-protection plans were optimized to meet the predefined rectal dose criteria in the absence of spacer-induced separation. Under setup and range perturbations, none of the robustness scenarios satisfied all prostate coverage criteria, resulting in a 0% prostate passing rate, whereas post-SpaceOAR plans achieved rectal sparing with improved robustness. This finding suggests that rectal-sparing strategies may compromise dose uniformity within the prostate target. Observed variations in composite scores were attributable to the ProKnow weighting structure. The ProKnow composite score used in this study was derived from a predefined, clinically informed weighting scheme in which each of the three rectum-related metrics was assigned a weight of 2, whereas all other metrics were assigned a weight of 1. Greater weight was assigned to rectum-related metrics to reflect the primary rationale for SpaceOAR use and the dosimetric priority of rectal sparing in prostate CIRT. As external validation of this weighting approach in CIRT is currently unavailable, the composite score should be interpreted as a relative comparative tool within the present analysis rather than as a universal measure of plan quality or a surrogate for clinical outcome.

In some patients, prioritization of rectal dose constraints was associated with reduced prostate subscores (Cases 6, 7, 18, and 21), whereas in others, exceptionally high rectal subscores contributed to comparatively elevated composite scores (e.g., Case 9). Overall, these results suggest that although manual rectal sparing can adversely affect PTV coverage, SpaceOAR insertion enables effective rectal dose reduction while preserving prostate dose homogeneity, thereby contributing to improved overall plan performance (**Figure 9**) (28).

In future prospective studies, a standardized bladder-filling protocol may further improve the stability and interpretability of ProKnow composite scoring by minimizing bladder-volume–related fluctuations in percentage-based DVH endpoints. More consistent bladder filling may reduce variability in bladder-related score components, thereby strengthening the robustness of composite plan-quality comparisons across plans and patients.

Comparing with the literature on photon-based radiotherapy, our findings appear to align with randomized controlled trials demonstrating that perirectal hydrogel spacers reduce rectal dose while preserving target coverage in prostate IMRT (13). Similarly, proton therapy studies have reported substantial reductions in rectal DVH parameters following SpaceOAR insertion across both passive-scattering and pencil-beam scanning modalities (29). Carbon ion beams exhibit elevated LET near the distal Bragg peak, which may influence biological effects in adjacent normal tissues. In this context, the present study extends existing spacer-related findings from photon and proton therapy into CIRT, a biologically and physically distinct modality characterized by a sharper lateral penumbra, higher LET and RBE, and greater sensitivity to anatomical variations, by demonstrating analogous dosimetric trends in this setting where geometric uncertainties may carry greater dosimetric and planning implications.

As the present analysis was based on clinically implemented Gy(RBE) distributions calculated with mMKM, the reported dosimetric differences reflect the RBE-weighted dose framework used in routine treatment planning, although separate LET-specific analysis was not performed.

A methodological strength of this study is its three-phase comparative design (Pre-SpaceOAR, Pre-SpaceOAR with rectum protection, and Post-SpaceOAR), which is not commonly reported in photon or proton studies. This design allowed better distinction between the effects of clinically implemented rectal-sparing strategies and the intrinsic geometric advantage introduced by spacer placement.

This study also provides a quantitative characterization of dose–geometry relationships by correlating prostate–rectum separation distance with rectal dosimetric indices (Vx, Dmean). This regression-based analysis offers one of the first numerical descriptions of translation of spacer-induced anatomical displacement into dose modulation in CIRT, a topic infrequently explored in photon or proton literature.

Its further strengths include the use of ProKnow scoring and robustness evaluation, which provide an objective framework for assessing plan quality. These analyses suggest that SpaceOAR improves rectal sparing and enhances plan stability and robustness, which is an important consideration in CIRT, where small anatomical variations can lead to amplified dosimetric effects due to range and LET sensitivities (**Table 2**, **6**; **Supplementary Table 3**; **Figures 4**, **8**, **9**).

As this study was based on treatment planning system calculations and simulation analysis, it evaluated the relative dosimetric, robustness, and plan-quality advantages associated with SpaceOAR insertion at the treatment planning stage by comparing calculated dose distributions before and after spacer placement. However, these findings alone cannot directly verify the accuracy of actual dose delivery. This consideration is particularly important in carbon ion radiotherapy, where dose distributions are highly sensitive to range uncertainty and tissue heterogeneity. Therefore, the present results should be interpreted as evidence suggesting relative dosimetric and planning advantages of SpaceOAR application, while further validation through phantom irradiation and ion chamber measurements is warranted to confirm concordance between calculated and delivered dose distributions (30, 31). This study was conducted as a retrospective planning and simulation analysis and did not include clinical endpoints, such as acute or late toxicity or patient-reported outcomes. Therefore, the observed dosimetric improvements and enhanced robustness should be interpreted as surrogate indicators and should not be assumed to translate directly into clinical benefit.

The small sample size of 25 patients may have reduced sensitivity for detecting smaller effects and may also limit the generalizability of the findings to broader patient populations or other institutional settings. Future studies with larger and more diverse cohorts will be important to confirm the present findings, detect more modest effects, and enable more stable estimation in exploratory or regression-based analyses.

In addition, real-world variability in patient anatomy and treatment delivery may introduce further complexities not fully captured in this dosimetric analysis. Thus, the findings should be regarded as exploratory and hypothesis-generating rather than definitive, with emphasis on identifying consistent dosimetric trends rather than making population-level inferences. Further studies in larger and more homogeneous patient populations are needed to validate and extend these results. Moreover, prospective study designs incorporating clinical outcome data will be essential to evaluate toxicity and quality-of-life metrics and to determine whether the observed reduction in rectal dose translates into meaningful clinical benefit for patients (32, 33).

## 5 Conclusions

In conclusion, this study demonstrates the association of SpaceOAR insertion with favorable dosimetric changes in CIRT plans for prostate cancer, including reduced rectal dose and preserved target coverage. The observed improvements in dose distribution and organ-at-risk sparing suggest that SpaceOAR may contribute to enhanced treatment plan quality in the CIRT setting.

Importantly, given the retrospective design and limited sample size of this study, these findings should be interpreted cautiously and viewed as preliminary. Further prospective studies with larger and more diverse patient cohorts, incorporating standardized toxicity assessments and patient-reported outcomes, will be essential to establish the clinical relevance of the observed dosimetric improvements. Within this context and pending additional validation, SpaceOAR may represent a promising adjunct for optimizing CIRT planning for prostate cancer.

## Data Availability

The data are not publicly available due to institutional privacy and ethical regulations. Requests to access the datasets should be directed to the corresponding author.
