# Supplementary material for: Comparative analysis of radiation therapy plans before and after biodegradable hydrogel (SpaceOAR) injection for reducing rectal toxicity in patients with prostate cancer undergoing carbon ion radiotherapy
Source: Front Oncol. 2026 Apr 20;16:1805204. doi: 10.3389/fonc.2026.1805204 (PMC13136013; doi:10.3389/fonc.2026.1805204)
Supplement: Supplementary file 1 [file SupplementaryFile1.pdf]

Supplementary Material

1 Supplementary Data

Figure S1

Sectional view illustrating the anatomical relationship between the prostate and rectum during SpaceOAR insertion under ultrasound guidance. The figure depicts the relative position of the hydrogel spacer and the corresponding approximate dose distribution before and after spacer injection, demonstrating the increased separation between the prostate and rectum achieved by SpaceOAR placement.

Figure S2

The scatter plots depict regression analyses between separation distance and selected rectal volume-based dose metrics and dose extremes. As shown in Figure S2A and S2B, rectal V15 and V50 demonstrated significant negative correlations with prostate–rectum separation ($p < 0.05$), indicating a reduction in rectal volume exposed to both low- and high-dose regions with increasing anatomical separation.

Dose-based parameters are presented in Figure S2C and S2D. Rectal minimum dose (Dmin) showed a significant decreasing trend with increasing separation distance ($p = 0.021$), whereas rectal maximum dose (Dmax) exhibited a decreasing tendency that did not reach statistical significance ($p = 0.140$). These supplementary analyses support the main findings by demonstrating consistent dose–geometry relationships across additional rectal DVH metrics, reinforcing the association between increased prostate–rectum separation and improved rectal dose sparing.

Table S1.

Volumes of the prostate (PTV), rectum, bladder, and penile bulb measured **for all 25 patients** with and without SpaceOAR insertion. Organ volumes were delineated on planning images and are presented in cubic centimeters (cm^3).

Table S2.

Rectal dose–volume histogram (DVH) parameters stratified by mean prostate–rectum separation distance. The table presents dose-based metrics (Dmin, Dmax, and Dmean) and volume-based parameters (V15, V20, V30, V40, and V50) for each patient. Separation distances are reported in centimeters (cm). DVH values are presented as percentages (%) and doses in gray (Gy).

Table S3.

Comparative ProKnow plan quality scores for pre-SpaceOAR, pre-SpaceOAR with rectum protection, and post-SpaceOAR treatment plans. Individual patient scores, percentage scores relative to the

maximum achievable score, and summary statistics (mean and standard deviation) are presented. A perfect ProKnow score corresponds to 64 points.

2 Supplementary Figures and Tables

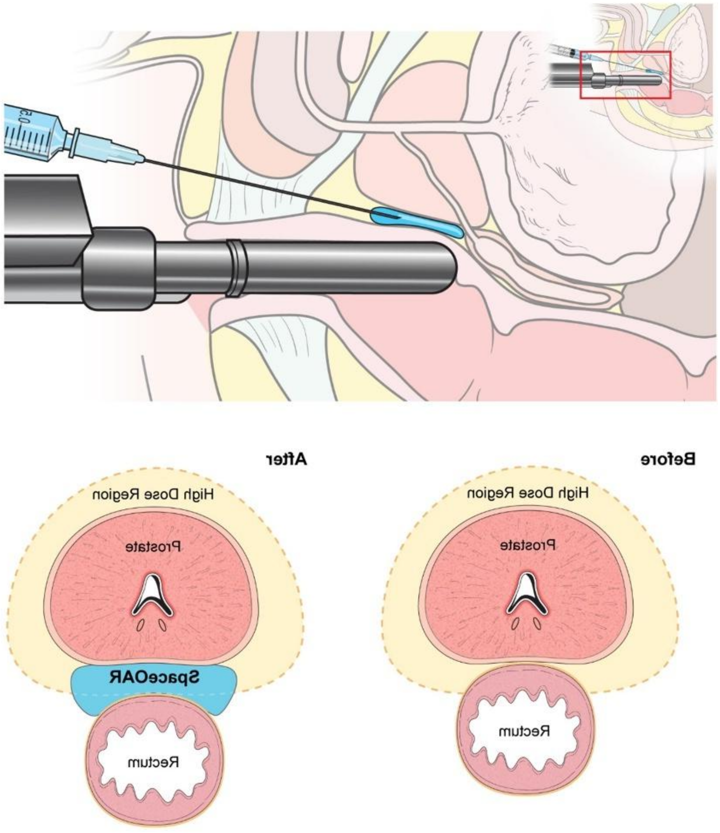

Figure S1 Sectional view of SpaceOAR insertion and dose distribution before and after injection. Abbreviations: OAR, organ at risk.

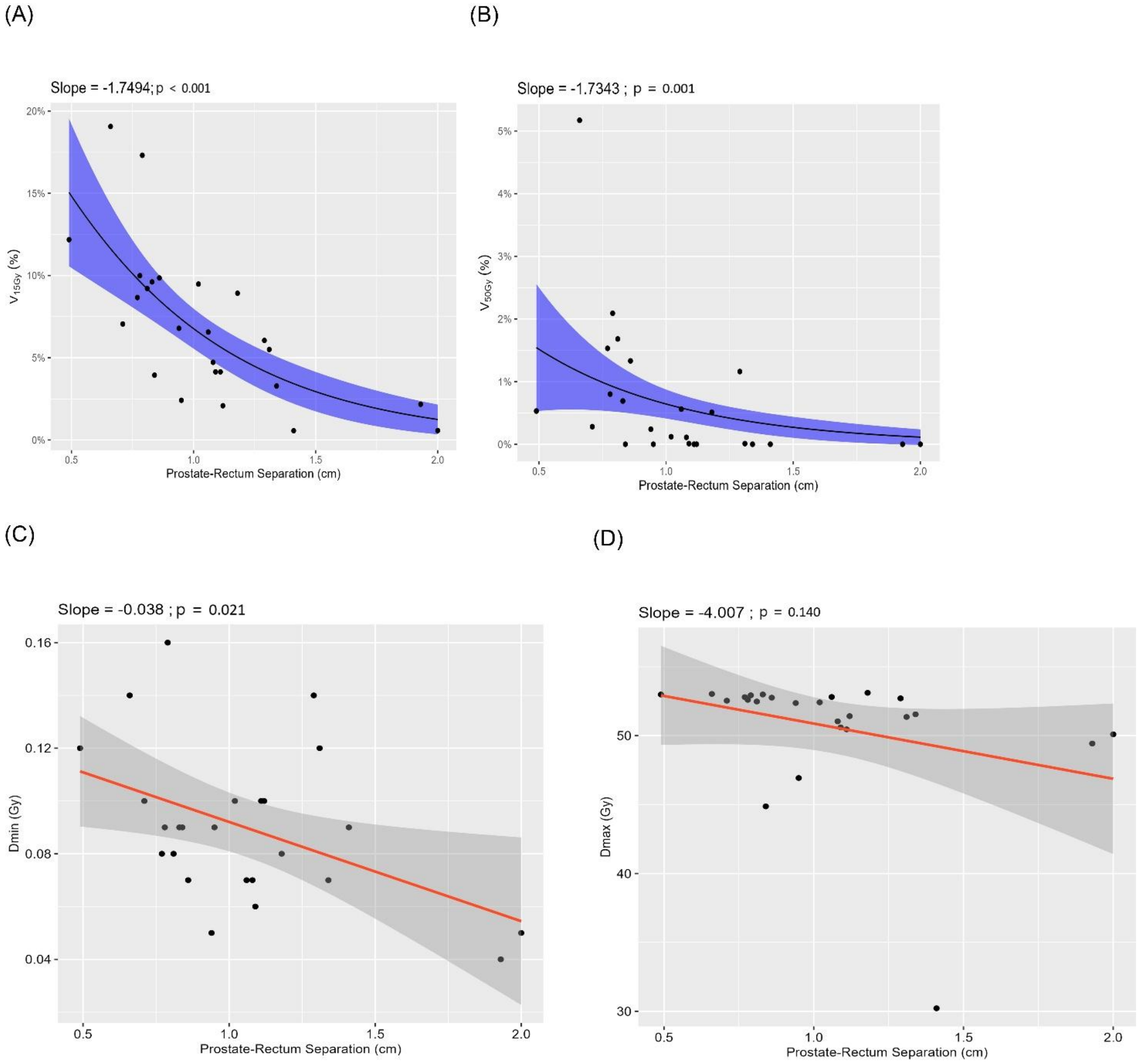

Figure S2 Rectum DVH values by Prostate-Rectum separation degree. (A) V_{15Gy} (%), (B) V_{50Gy} (%), (C) Dmin (Gy), and (D) Dmax (Gy).

Table S1 Volumes of the Target and surrounding organs (With/Without SpaceOAR).

Patient No.	PTV (Prostate) (cm³)	Rectum (cm³)	Bladder (cm³)	Penile Bulb (cm³)
	with/without	with/without	with/without	with/without
1	30.88 / 34.30	43.37 / 45.31	343.01 / 109.38	7.25 / 7.15

2	38.08 / 40.83	22.42 / 32.04	340.97 / 198.39	7.89 / 7.88
3	48.54 / 50.91	43.09 / 51.91	265.41 / 221.96	5.81 / 5.53
4	45.63 / 51.20	38.06 / 41.71	323.51 / 45.74	7.48 / 6.56
5	51.91 / 56.47	63.21 / 68.77	196.83 / 372.11	8.22 / 8.67
6	37.00 / 36.64	39.96 / 45.89	143.79 / 238.22	5.55 / 5.80
7	49.15 / 54.55	38.89 / 48.88	417.09 / 120.99	6.49 / 7.25
8	32.30 / 35.50	53.49 / 58.73	407.21 / 176.06	6.52 / 6.21
9	72.14 / 66.50	55.25 / 86.27	269.25 / 161.41	5.64 / 5.54
10	58.98 / 51.54	44.10 / 50.52	273.02 / 148.13	3.89 / 3.79
11	25.43 / 28.80	31.72 / 29.23	493.41 / 113.27	3.26 / 3.30
12	32.79 / 34.62	54.03 / 75.52	363.67 / 853.07	5.64 / 5.50
13	39.16 / 42.42	46.20 / 38.62	249.03 / 93.59	4.98 / 5.19
14	39.43 / 44.93	31.52 / 57.04	309.19 / 86.95	9.38 / 9.16
15	52.63 / 48.07	42.13 / 50.81	255.04 / 40.19	6.86 / 7.10
16	55.14 / 59.64	56.30 / 93.50	261.00 / 120.86	7.74 / 8.10
17	49.19 / 49.38	40.13 / 56.16	186.62 / 64.54	5.35 / 5.64
18	43.83 / 46.34	63.25 / 48.65	381.88 / 145.87	9.10 / 9.48
19	25.61 / 27.04	55.77 / 65.29	121.18 / 136.51	10.01 / 9.86
20	58.89 / 55.40	41.10 / 36.38	179.53 / 100.53	5.32 / 5.54

21	26.81 / 25.97	37.16 / 28.03	196.76 / 78.28	4.09 / 4.27
22	50.74 / 49.26	52.00 / 58.45	218.07 / 293.29	7.96 / 8.00
23	36.66 / 37.34	44.78 / 54.17	184.76 / 96.50	8.52 / 8.26
24	67.37 / 68.39	41.96 / 56.31	205.15 / 419.99	7.58 / 7.87
25	60.98 / 67.70	38.10 / 51.48	223.60 / 124.56	8.16 / 7.77

Note: Volumes were measured in cubic centimeters (cm^3). The data represent the volumes with and without SpaceOAR for each patient.

Table S2 Rectum DVH values by Prostate-Rectum separation degree.

Patient No.	Separation Mean (cm)	Dmin (Gy)	Dmax (Gy)	Dmean (Gy)	V_{15} (%)	V_{20} (%)	V_{30} (%)	V_{40} (%)	V_{50} (%)
1	0.81	0.08	52.48	4.23	9.21	7.28	5.68	3.48	1.68
2	0.66	0.14	53.03	8.46	19.06	15.46	13.26	9.19	5.17
3	0.77	0.08	52.80	4.12	8.66	7.10	5.20	3.42	1.53
4	0.83	0.09	52.99	4.19	9.61	6.98	5.07	2.66	0.69
5	1.34	0.07	51.55	1.77	3.28	2.13	0.78	0.17	0.00
6	2.00	0.05	50.10	0.73	0.57	0.42	0.15	0.03	0.00
7	1.18	0.08	53.11	4.16	8.92	6.86	4.18	2.14	0.51
8	1.06	0.07	52.81	3.07	6.56	5.06	3.05	1.72	0.56
9	0.79	0.16	52.93	7.62	17.31	14.37	10.59	6.70	2.09
10	1.02	0.10	52.42	4.14	9.48	7.04	3.80	1.60	0.12

11	1.08	0.07	51.04	2.33	4.72	3.29	2.06	0.57	0.11
12	0.95	0.09	46.93	1.55	2.41	1.34	0.45	0.04	0.00
13	0.94	0.05	52.37	3.14	6.79	5.44	3.44	1.76	0.24
14	1.31	0.12	51.36	2.75	5.50	4.16	1.91	0.50	0.01
15	0.71	0.10	52.54	3.29	7.05	5.17	3.28	1.53	0.28
16	0.86	0.07	52.76	4.48	9.85	8.02	5.55	3.52	1.33
17	1.41	0.09	30.22	1.10	0.56	0.12	0.00	0.00	0.00
18	1.93	0.04	49.43	1.33	2.17	1.58	0.66	0.18	0.00
19	1.09	0.06	50.60	2.00	4.14	3.18	1.68	0.62	0.01
20	0.49	0.12	52.99	5.15	12.18	8.35	6.97	3.22	0.53
21	0.78	0.09	52.60	4.45	9.99	7.80	5.28	2.99	0.80
22	0.84	0.09	44.87	1.93	3.94	2.22	0.72	0.07	0.00
23	1.12	0.10	51.42	1.54	2.08	1.54	0.45	0.19	0.00
24	1.29	0.14	52.71	3.11	6.05	4.98	3.79	2.55	1.16
25	1.11	0.10	50.46	2.32	4.14	2.93	1.22	0.41	0.00

Note: The mean separation value is measured in centimeters (cm). DVH values are presented as percentages (%) and doses in gray (Gy). The data were rounded to two decimal places.

Table S3 Comparative Analysis of Plan Scores Pre-SpaceOAR, Pre-SpaceOAR (Rectum Protection), and Post-SpaceOAR insertion.

Case	Pre-SpaceOAR	%	Pre-SpaceOAR (Rectum Protection)	%	Post- SpaceOAR	%
1	27.09	42.33%	29.27	45.73%	53.16	83.06%
2	36.17	56.52%	38.10	59.53%	46.82	73.16%
3	25.77	40.27%	31.92	49.88%	54.15	84.61%
4	20.68	32.31%	25.06	39.16%	58.49	91.39%
5	27.42	42.84%	36.08	56.38%	58.91	92.05%
6	37.66	58.84%	35.93	56.14%	60.00	93.75%
7	28.37	44.33%	26.66	41.66%	57.78	90.28%
8	25.10	39.22%	35.27	55.11%	61.51	96.11%
9	22.01	34.39%	43.03	67.23%	37.63	58.80%
10	29.03	45.36%	37.35	58.36%	58.57	91.52%
11	37.77	59.02%	41.26	64.47%	59.65	93.20%
12	28.41	44.39%	34.00	53.13%	59.40	92.81%
13	25.69	40.14%	32.73	51.14%	58.58	91.53%
14	22.13	34.58%	31.15	48.67%	59.52	93.00%
15	34.77	54.33%	45.34	70.84%	60.30	94.22%
16	23.74	37.09%	32.38	50.59%	51.30	80.16%

17	24.10	37.66%	27.31	42.67%	59.27	92.61%
18	31.54	49.28%	29.18	45.59%	59.75	93.36%
19	24.00	37.50%	31.69	49.52%	59.72	93.31%
20	27.53	43.02%	33.14	51.78%	56.78	88.72%
21	36.75	57.42%	35.82	55.97%	58.73	91.77%
22	23.49	36.70%	31.01	48.45%	58.11	90.80%
23	29.06	45.41%	34.19	53.42%	59.96	93.69%
24	27.45	42.89%	36.22	56.59%	56.44	88.19%
25	29.05	45.39%	30.85	48.20%	59.96	93.69%
Mean	28.19	44.05%	33.80	52.81%	56.98	89.03%
SD	5.03	7.86%	4.89	7.64%	5.19	8.11%

Note: A perfect score in this analysis is 64 points.
